# Evaluating the Feasibility of Low-Cost, Contactless Consumer Sleep-Tracking Devices as Measurement Tools for Preliminary Sleep Research

**DOI:** 10.3390/s26082371

**Published:** 2026-04-12

**Authors:** Huifang Zhai, Yonghong Yan, Litao Gao, Siqi He, Xiaowan Dong, Xiang Cheng, Tao Hu

**Affiliations:** 1 School of Architecture and Urban Planning, Chongqing University, Chongqing 400044, China; zhaihuifang@cqu.edu.cn (H.Z.); chengxiang@cqu.edu.cn (X.C.); 20161501009@cqu.edu.cn (T.H.); 2 Key Laboratory of New Technology for Construction of Cities in Mountain Area, Chongqing University, Chongqing 400044, China; 3 School of Electrical and Electronic Engineering, Chongqing Vocational and Technical University of Mechatronics, Chongqing 402760, China; gaolitao@cqvtu.edu.cn; 4 College of Landscape Architecture, Nanjing Forestry University, Nanjing 210037, China; hesiqi@njfu.edu.cn; 5 School of Architecture and Urban Planning, Henan University of Urban Construction, Pingdingshan 467036, China; dxiaowan@huuc.edu.cn

**Keywords:** sleep, actigraphy, contactless, validation, sensors, polysomnography

## Abstract

**Highlights:**

**What are the main findings?**

- For all sleep metrics except sleep stages, the iSleep S200G and Sleep Dot B501 showed no significant differences compared to PSG.
- The iSleep S200G and Sleep Dot B501 demonstrate comparable or even superior reliability levels to Actiwatch Spectrum in sleep/wake measurements and EBE agreement.

**What are the implications of the main findings?**

- This study offers a potential solution for conducting long-term, large-scale preliminary sleep research with low precision requirements under budget constraints.
- The performance validation results provide data references for researchers adopting iteratively upgraded CCSTDs.

**Abstract:**

Compared to polysomnography (PSG) and actigraphy, contactless consumer sleep-tracking devices (CCSTDs) are low-cost, user-friendly, and non-disruptive to sleep. This study evaluated the performance of two inexpensive, representative first-generation Chinese-made CCSTDs (the iSleep S200G and Sleep Dot B501) against PSG and actigraphy, using standardized validation protocols. The objective was to assess their feasibility as alternatives for large-scale, long-term preliminary research that does not rely on single-day high-precision sleep data. Eleven healthy young adults (mean age = 26.5 ± 4.8 years) participated in a two-night sleep laboratory study using four devices in parallel. Compared with PSG, the iSleep S200G exhibited no significant differences in TST and SE, while the Sleep Dot B501 showed no significant differences in TST, SE, SOL, and WASO. The intraclass correlation coefficient values and epoch-by-epoch agreement of the iSleep S200G and Sleep Dot B501 were as good as or better than those of actigraphy. Notably, the epoch-by-epoch agreement metric of both devices was not inferior to other consumer sleep-tracking devices already used for long-term, large-scale sleep monitoring. Therefore, even within budget constraints, first-generation CCSTDs can effectively meet the requirements for long-term, large-scale sleep monitoring without sleep stage detection. The results also provided data references for researchers using iteratively upgraded CCSTDs.

## 1. Introduction

Long-term, large-scale data collection for preliminary sleep assessments is constrained by the high costs and specialized operational requirements of gold-standard polysomnography (PSG) and silver-standard actigraphy. This limitation is particularly pronounced in economically underdeveloped regions with low education levels and poor living conditions. As a result, their widespread use in such settings is often impractical. To address these unique scenarios, exploring a more suitable monitoring alternative is urgently needed. Such a device should be low-cost, easy to operate, and user-friendly for data processing, with strong environmental adaptability for stable operation in rudimentary conditions. Consumer sleep-tracking devices offer a viable approach, as recent technological advancements have reduced the performance disparity between consumer-grade and research-grade devices [1,2]. Numerous validation studies, using recognized validation standards [3,4,5], have also confirmed that many consumer sleep-tracking devices have achieved or surpassed the accuracy of actigraphy [6,7,8,9,10]. Several validated commercial sleep devices, including Huawei FIT 2 [11], Withings sleep analyzer [12], Readiband [8], HSL-102M [13], and SleepScore Max [8], have been extensively utilized in sleep monitoring experiments in various fields such as healthful lighting [14,15], healthcare [16,17], and sports [18,19,20]. These application results indicate that consumer sleep-tracking devices have unparalleled advantages in large-scale and long-term sleep monitoring and tracking.

Consumer sleep-tracking devices generate massive datasets of sleep parameters through continuous passive sleep tracking. These datasets enable quantification of sleep and circadian rhythm metrics, supporting large-scale preliminary assessments of sleep duration, timing, efficiency, quality, patterns, and regularity [21,22,23]. The expert consensus from the 2018 International Biomarkers Workshop on Wearables in Sleep and Circadian Science stated that device performance should be judged by its intended application [21]. For large population studies, lower sensitivity/specificity is acceptable; however, smaller laboratory studies or clinical use in individual patients require the best possible device performance. Therefore, low-cost consumer sleep-tracking devices that do not rely on single-day high-precision sleep data exhibit great potential for use in large-scale, long-term preliminary sleep research.

Nevertheless, researchers face a complicated selection process. The consumer sleep-tracking device market features a wide variety of products with significant price differences, and its rapid product update cycle far outpaces the pace of validation studies [22]. At the same time, considering that real-world usage scenarios may involve certain vulnerable and sensitive individuals, such as burn victims, infants, and psychiatric patients, wearable consumer sleep-tracking devices may still cause interference [23,24,25,26]. Therefore, this study focused on contactless consumer sleep-tracking devices (CCSTDs) and validated two representative low-cost first-generation models in the Chinese market: the iSleep S200G and Sleep Dot B501. Based on the validation results, the feasibility of using low-cost CCSTDs based on first-generation technology for long-term, large-scale preliminary sleep studies was evaluated. Simultaneously, it offered performance benchmarks for researchers using iteratively upgraded CCSTDs. The validation experiment followed the research reports and guidelines for sleep device performance proposed by the Sleep Research Society and Menghini et al. [4,21].

## 2. Materials and Methods

### 2.1. Participants

In this study, a total of 11 healthy young adults, including 6 men and 5 women, with an average age of 26.5 ± 4.8 years (mean ± SD) and a body mass index calculated based on height and weight of 21.9 ± 2.3 kg/m^2^ (mean ± SD), were included as participants. These individuals had no neurological or psychiatric diagnoses, sleep disorders, or other medical conditions. Alcohol, caffeine, and any drugs were strictly prohibited during the research. Participants were recruited at Chongqing University between June and July 2020. This study strictly adhered to the ethical guidelines of the Declaration of Helsinki and has been approved by the Ethics Committee of South China Normal University (No. SCNU-AOE-2025-018). This is a subsequent administrative approval for reanalyzing previously collected data. All participants voluntarily signed written informed consent forms after receiving complete disclosure.

### 2.2. Sleep Monitors

#### 2.2.1. Contactless Consumer Sleep-Tracking Devices

For this study, we selected two representative CCSTDs. The iSleep S200G, recognized as the first CCSTD launched in China [27], was developed by XIKANG ALPS Technology Co., Ltd. (Shenyang, China) and released in 2014 at a price of $180 [28]. The Sleep Dot B501, the affordable CCSTD (priced below $14), was manufactured by Sleepace Co., Ltd. (Shenzhen, China) and introduced in 2015 [29].

The iSleep S200G uses a 2.45 GHz radio-frequency micro-power radar to monitor body movement and respiration [30]. The sleep monitoring device was placed approximately 0.5 to 0.8 m from the body, oriented toward the chest. Sleep data could be accessed and monitored via the display screen and the associated application. The monitored sleep parameters include total time in bed (TIB), total sleep time (TST), total wake time (TWT), sleep efficiency (SE), light sleep (N1), moderate sleep (N2), and deep sleep (N3).

The Sleep Dot B501 is equipped with a magnetic clasp designed for attachment to the edge of a pillow. The device uses a 3-axis accelerometer to monitor sleep patterns by tracking body movements. It connects to a mobile application through Bluetooth, enabling users to access and analyze their sleep data. The sleep parameters that can be directly measured include TIB, TST, TWT, SE, and the various stages of sleep, such as N1, N2, and N3. Wake after sleep onset (WASO) and sleep onset latency (SOL) can be calculated using the recorded bedtime, sleep onset time, and TWT provided by the device. We also contacted Sleepace to obtain the original 60 s epoch data. Light sleep, moderate sleep, and deep sleep in both the iSleep S200G and Sleep Dot B501 correspond to the N1, N2, and N3 stages in PSG, respectively.

During sleep stage assessment, although the two devices are capable of monitoring the indicators of the three non-rapid eye movement sleep sub-stages (N1, N2, and N3), they do not support the rapid eye movement.

#### 2.2.2. PSG and Actigraphy

We used the SOMNOtouch™ RESP (SOMNOmedics GmbH & Co. KG, Randersacker, Germany), which is currently the most compact full-color touchscreen PSG device available on the market. This device was utilized to collect sleep data from various sources: electroencephalography (recorded at F3, C3, O1, Fpz, and Cz sites with reference to the contralateral mastoids), bilateral electrooculography, mentalis electromyography, tibialis anterior electromyography, pulse oximetry, electrocardiography, and a respiratory monitoring sensor. At the beginning of the recordings, impedances were equal to or less than 10 kΩ. PSG electrode sites were measured and applied according to standard criteria, specifically following the International 10–20 System of Electrode Placement.

PSG sleep stages, including N1, N2, N3, and rapid eye movement (REM), as well as arousals, were manually assessed with the assistance of an experienced sleep technologist. The sleep summary and epoch-by-epoch (EBE) analysis employed the conventional 30 s PSG scoring epoch.

Participants used the Actiwatch Spectrum, a research-grade actigraphy device manufactured by Philips Respironics, Inc., in Murrysville, PA, USA. Participants were instructed to wear the actigraphy device on their non-dominant wrist throughout the entire night. We configured the devices to operate at a medium-threshold sensitivity of 40 activity counts per epoch and collected sleep data in 30 s epochs. Actiwatch Spectrum provided a binary sleep versus wake classification (0 = Sleep, 1 = Wake).

### 2.3. Procedures

Sleep data was collected from each participant over two nights in a sleep laboratory that had been converted from an apartment at Chongqing University. This data collection was conducted from August to September 2020. The laboratory replicated the sleeping environment typically found in a home. Only one participant was scheduled for the experiment each night, and all participants used the same equipment. Before the formal assessment, every participant underwent a period of acclimatization with the PSG device, lasting one to two nights. Participants arrived at the laboratory two hours before their usual bedtime. The researcher installed the PSG electrodes, configured the Actiwatch Spectrum, and set up the iSleep S200G and Sleep Dot B501 devices. The Actiwatch Spectrum activated automatically when worn, while the other devices activated simultaneously before lights out. Participants were instructed to follow their usual sleep routines, going to bed and waking up at their regular times. Upon waking, the participant turned on the lights, after which researchers entered to assist with device deactivation and electrode removal. TIB for all devices was calculated based on the lights-out and lights-on times. The experimental study was conducted under strict supervision without any intervention. The outcome measures included TST, SOL, WASO, SE, light sleep (N1 + N2), and deep sleep (N3).

### 2.4. Statistical Analyses

Sleep data collected from the iSleep S200G, Sleep Dot B501, and Actiwatch Spectrum devices were compared to PSG recordings. Statistically significant differences between each device and PSG were assessed using paired *t*-tests or Wilcoxon signed-rank tests, with corresponding *p*-values reported. The normality of the data was evaluated by conducting the Shapiro–Wilk test. For all continuous variables that adhered to a normal distribution, the mean and standard deviation (SD) were provided, and paired *t*-tests were used. For variables exhibiting a non-normal distribution, the median and interquartile range (IQR) were reported, and the Wilcoxon signed-rank test was used. *p*-values that fell below the threshold of *p* < 0.05 were considered statistically significant. Hedges’ g was reported as the effect size, which provides an unbiased estimate of the standardized mean difference, especially advantageous in small-sample studies compared to Cohen’s d [31]. Interpretation of g is 0.2 (small effect), 0.5 (medium effect), and 0.8 (large effect) [32]. Absolute agreement and reliability between the PSG and each of the selected testing devices were evaluated using a two-way random effects model to calculate the intraclass correlation coefficient (ICC). ICC values indicate poor agreement for less than 0.50, moderate agreement for between 0.50 and 0.75, good agreement for between 0.75 and 0.90, and excellent agreement for greater than 0.90 [33].

A Bland–Altman plot was employed to visually represent the level of agreement between PSG and each of the chosen devices. The meaning of two measurements was plotted on the x-axis, and the discrepancy between them was plotted along the y-axis. Calculating the mean difference estimated the average deviation between PSG and other devices. The lower and upper limits of agreement (LOA) were determined by calculating the mean difference ± 1.96 SD. LOA is a statistical method used to estimate the range within which a specific proportion of differences between measurements is expected to fall.

The present study employed EBE agreement statistics to assess the agreement between sleep and wake epochs recorded by each device in relation to the corresponding epochs scored by PSG. These statistics include sensitivity (true positive rate), specificity (true negative rate), positive predictive value (PPV), negative predictive value (NPV), and accuracy. Sensitivity refers to the proportion of sleep epochs correctly detected as sleep by the PSG device, while specificity represents the proportion of wake epochs correctly detected as wake by the PSG device. PPV indicates the proportion of sleep epochs scored by the device that were accurately identified as true PSG sleep, while NPV represents the proportion of wake epochs scored by the device that were accurately identified as true PSG wake. Accuracy, on the other hand, refers to the proportion of all PSG epochs correctly detected by the device [4].

All data were analyzed using IBM SPSS 20. GraphPad Prism 6 software was utilized to conduct Bland–Altman analyses to assess the level of agreement between two devices for each sleep parameter.

### 2.5. Missing Data Procedures

**Figure 1:**
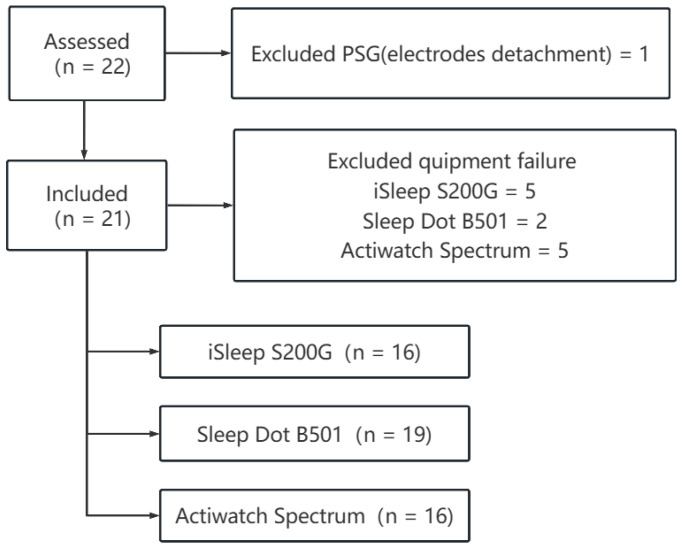
Study flow diagram.

At each lab visit, the researchers carefully synchronized all devices and charged the Actiwatch Spectrum to collect sleep data from PSG, actigraphy, and CCSTDs during scheduled sleep periods. However, on one night, the polysomnography electrodes detached while the subject was asleep, which led to the exclusion of all monitoring device data for that specific night. Additionally, due to device malfunctions, data from five nights of iSleep S200G reports, two nights of Sleepace reports, and five nights of Actiwatch Spectrum reports were excluded from the analysis (Figure 1). All paired comparisons were restricted to the same retained nights for each device.

## 3. Results

### 3.1. Sleep Summary Outcomes

**Figure 2:**
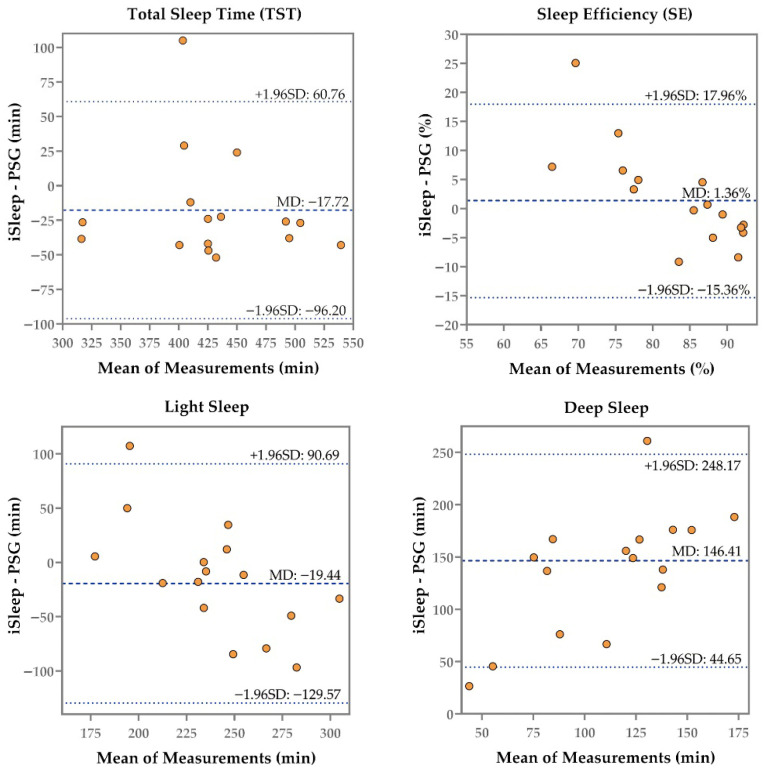
Bland–Altman plot illustrating the comparison of four outcomes (TST, SE, Light Sleep, and Deep Sleep) derived from the iSleep S200G and PSG recordings. The middle line represents the mean difference, while the upper and lower dashed lines indicate the upper and lower limits of agreement (mean difference ± 1.96 SD).

**Figure 3:**
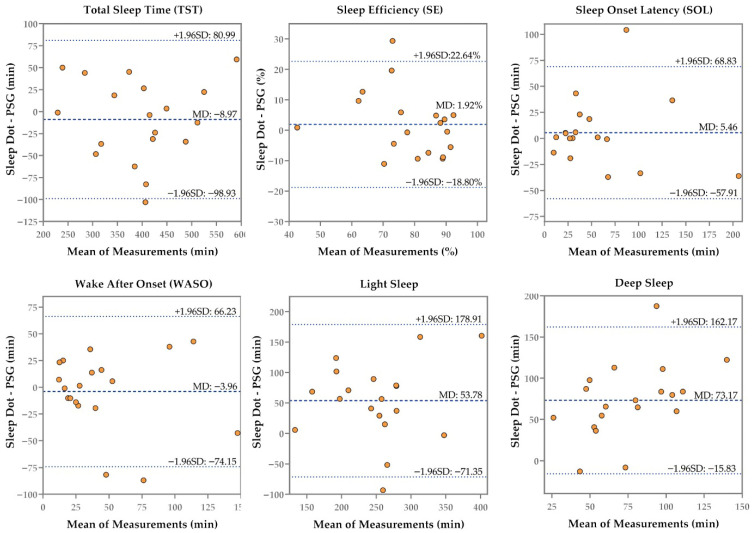
Bland–Altman plot of the six outcomes (TST, SE, SOL, WASO, Light Sleep, and Deep Sleep) recorded by the Sleep Dot B501 and PSG. The middle line represents the mean difference, while the upper and lower dashed lines indicate the upper and lower limits of agreement (mean difference ± 1.96 SD).

**Figure 4:**
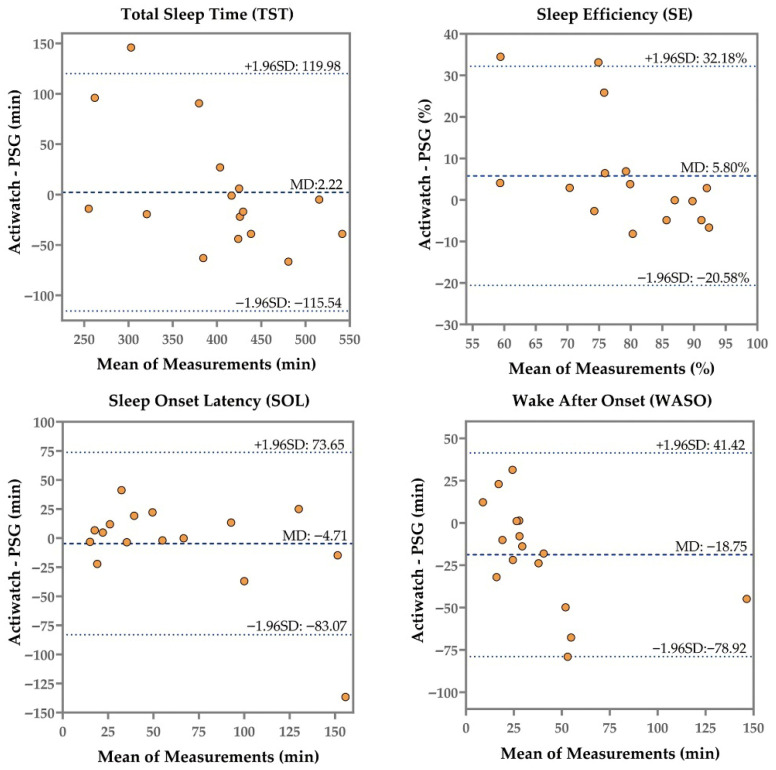
Bland–Altman plot of the four outcomes (TST, SE, SOL, WASO) recorded by the Actiwatch Spectrum and PSG. The middle line represents the mean difference, while the upper and lower dashed lines indicate the upper and lower limits of agreement (mean difference ± 1.96 SD).

Table 1 displays the summary measures of the iSleep S200G, Sleep Dot B501, and Actiwatch Spectrum compared to PSG, and Figure 2, Figure 3 and Figure 4 present the corresponding Bland–Altman plots.

Despite overestimating or underestimating TST, SE, SOL, and WASO, the iSleep S200G, Sleep Dot B501, and Actiwatch Spectrum showed no significant differences compared to PSG—except for the Actiwatch Spectrum’s WASO (*p* = 0.04, Hedges’ g = 0.53). The iSleep S200G significantly overestimated deep sleep (*p* < 0.001, Hedges’ g = −3.53), while the Sleep Dot B501 significantly overestimated both light sleep (*p* = 0.002, Hedges’ g = −0.76) and deep sleep (*p* < 0.001, Hedges’ g = −1.99). However, the iSleep S200G underestimated light sleep by an average of 19.44 min, showing no significant difference compared with PSG. As shown in Figure 2, the Bland–Altman plot indicated that as the mean duration of light sleep measurements increased, the iSleep S200G shifted from overestimation to underestimation.

Compared with PSG, the iSleep S200G, Sleep Dot B501, and Actiwatch Spectrum showed good agreement in measuring TST, with ICC values of 0.78, 0.89, and 0.77, respectively. The Sleep Dot B501 demonstrated moderate agreement for SE (ICC = 0.71), while the iSleep S200G (ICC = 0.48) and Actiwatch Spectrum (ICC = 0.38) exhibited poor agreement. The Actiwatch Spectrum exhibited lower agreement in both TST and SE compared with the iSleep S200G and Sleep Dot B501. The Bland–Altman plots for SE from the iSleep S200G (Figure 2), Sleep Dot B501 (Figure 3), and Actiwatch Spectrum (Figure 4) indicated that when subjects exhibited higher mean SE values, the biases of each device relative to PSG decreased. Sleep Dot B501 demonstrated better agreement than the Actiwatch Spectrum in SOL and WASO. As illustrated in Figure 3 and Figure 4, the Bland–Altman plots for SOL and WASO showed that when participants had lower SOL and WASO values, the biases from PSG exhibited the smallest magnitude and least variability. The iSleep S200G and Sleep Dot B501 both exhibited poor agreement in light and deep sleep.

### 3.2. EBE Analysis Outcomes

The level of agreement between EBE in sleep and wake states, as compared with PSG for all nights, is displayed in Table 2.

The sensitivity of the iSleep S200G (0.91) and Sleep Dot B501 (0.94) compared to PSG was found to be excellent. Sleep Dot B501 exhibited higher sensitivity compared to Actiwatch Spectrum (0.92), whereas iSleep S200G demonstrated slightly lower sensitivity. However, the specificity of the two CCSTDs (iSleep S200G = 0.65, Sleep Dot B501 = 0.68) was lower than their sensitivity. Although both devices showed lower accuracy in detecting wake epochs compared with sleep epochs, they outperformed the Actiwatch Spectrum (0.58).

Other indicators of agreement between EBE and PSG, as presented in Table 2, primarily aligned with the results of sensitivity and specificity. The PPV for both devices (iSleep S200G = 0.92, Actiwatch Spectrum = 0.91) was high. This finding implied that the sleep epochs recorded by these devices accurately represented sleep as assessed by PSG. The NPV for both the iSleep S200G (0.49) and the Actiwatch Spectrum (0.57) was relatively lower, indicating that the device-scored wake epochs poorly reflected PSG scoring compared with device-scored sleep epochs. Sleep Dot B501 demonstrated a PPV of 0.91 and the highest NPV of 0.74. All devices demonstrated a similar level of accuracy.

## 4. Discussion

### 4.1. Principal Results

Overall, although the iSleep S200G and Sleep Dot B501 are affordable and have been on the market for over a decade, their performance remains commendable compared to PSG and actigraphy. Compared to PSG, the iSleep S200G showed statistically significant differences in deep sleep, the Sleep Dot B501 in both light and deep sleep stages, and the Actiwatch Spectrum in WASO. The EBE analysis results indicated that the iSleep S200G and Sleep Dot B501 demonstrated superior performance in sleep detection. They exhibited higher sensitivity in detecting sleep compared to wake, but relatively low specificity when compared to PSG. However, the accuracy of both devices was found to be lower during the sleep stages. This result is consistent with the validation conclusions of consumer sleep devices such as Garmin Fenix 5S, Garmin Vivosmart 3, Earlysense Live [8], Somnofy [34], and the Beddit Sleep Tracker [35]. This indicates that two devices exhibit relatively low reliability for monitoring light sleep and deep sleep. The iSleep S200G demonstrated comparable levels of accuracy, sensitivity, and specificity to actigraphy. The Sleep Dot B501 exhibited superior accuracy and sensitivity compared to actigraphy, although it had a slightly lower level of specificity. The findings also demonstrated a high level of agreement for TST in iSleep S200G, as well as TST and SOL in Sleep Dot B501. The agreement was moderate for SE and WASO in the Sleep Dot B501. The agreement levels of Actiwatch Spectrum were inferior to those of both iSleep S200G and Sleep Dot B501 regarding TST, SE, SOL, and WASO. The iSleep S200G and Sleep Dot B501 demonstrated performance comparable to, and even superior to, the Actiwatch Spectrum in terms of EBE agreement in sleep and wake.

### 4.2. Comparison with Prior Work

In comparison to other CCSTDs with radar sensors, such as the ResMed S+ (accuracy: 0.88, sensitivity: 0.93, specificity: 0.51) [8], SleepScore Max (accuracy: 0.88, sensitivity: 0.94, specificity: 0.50) [8], Somnofy (accuracy: 0.76, sensitivity: 0.97, specificity: 0.72) [34], SleepMinder (accuracy: 0.77, sensitivity: 0.86, specificity: 0.52) [36], the iSleep S200G consistently showed strong performance in terms of EBE agreement. We compared the EBE agreement of the Sleep Dot B501 with other accelerometer-based consumer sleep devices. These included the non-sleep staging Fitbit (accuracy: 0.81–0.91, sensitivity: 0.87–0.99, specificity: 0.10–0.52), the sleep staging Fitbit model (accuracy: 0.90, sensitivity: 0.95–0.96, specificity: 0.58–0.69) [2], and the ActiGraph GT3X+ (accuracy: 0.89, sensitivity: 0.97, specificity: 0.24–0.25) [37]. The Sleep Dot B501 exhibited outstanding capabilities in sleep epoch identification.

The comparative analysis above demonstrated that both the iSleep S200G and Sleep Dot B501 exhibited high reliability in sleep monitoring. However, their suitability as experimental instruments across different sleep research scenarios could be evaluated by comparing them with other consumer-grade sleep monitoring devices already used in experiments.

Research Scenario 1: Long-term Sleep Monitoring. Among existing research, wristband-style sleep monitoring devices were the most widely used. We selected three high-performance devices for comparative analysis. POWER C J et al. [20] observed the sleep patterns of seven semi-professional female basketball players over a 13-week duration utilizing wrist-worn sleep monitors, Readiband™ (accuracy: 0.88, sensitivity: 0.94, specificity: 0.45) [8]. The sleep metrics included in the quantitative study were time in bed, TST, WASO, SE, sleep onset, and sleep offset (the time of day when last waking before getting up). Readiband was utilized in an additional sleep intervention trial focused on enhancing sleep, mood, and cognitive function in athletes. The research gathered sleep monitoring data from 56 e-sports participants across Korea, the United States, and Australia during a period of four weeks preceding and following the intervention. The evaluated sleep parameters comprised sleep onset time, TST, SOL, WASO, SE, time in bed, and wake-up time [38]. Compared with Readiband, Sleep Dot B501 exhibited superior performance across all sleep metrics necessary for the experiment and showed greater agreement in EBE. But the iSleep S200G had slightly lower accuracy and sensitivity, and it could not offer data for SOL or WASO. Fritz H et al. [39] performed a study examining the influence of indoor air quality on sleep quality with the Fitbit Inspire HR. The Fitbit exhibited an accuracy of 0.89, a sensitivity of 0.93, and a specificity of 0.45 [9]. The research gathered sleep data from 20 people over 77 days, encompassing assessments of TST, SE, REM, and non-rapid eye movement (NREM). Both the iSleep S200G and Sleep Dot B501 fulfilled the experiment’s requirements; however, the Sleep Dot B501 surpassed the Fitbit Inspire HR. A separate study investigating the effects of home isolation during the COVID-19 epidemic on sleep data employed the Garmin Fenix 6 Pro [40]. The research gathered sleep data, encompassing TST, light sleep, deep sleep, SOL, and REM, from 16 male professional fitness trainers over a duration of four consecutive months. The Garmin Fenix 6 Pro selected for this investigation was predicated on the validation outcomes of the Garmin Fenix 5S (accuracy: 0.87, sensitivity: 0.56, specificity: 0.92) [8], which notably excelled in wakefulness monitoring but exhibited inferior sleep-tracking skills relative to the iSleep S200G and Sleep Dot B501. Neither the iSleep S200G nor the Sleep Dot B501 possessed the capability to track REM sleep.

Experimental Scenario 2: Sleep Monitoring in Large or Global Samples. Hirata T. et al. [17] utilized the HSL-102M device (HSL-101: accuracy: 0.588, sensitivity: 0.963, specificity: 0.376) [13] to evaluate the population attributable fraction of home hypertension. A large-scale sleep monitoring study was conducted over 10 days, involving 1474 participants. The iSleep S200G and Sleep Dot B501 were superior to the HSL-101 in terms of accuracy and specificity, and they only had slightly lower sensitivity. Another large-scale study examined the impact of smartphone usage on adolescents’ sleep duration, involving 614 participants who provided TST data from 17 validated consumer-grade wearable sleep monitoring devices [41]. The Xiaomi Mi-Band (accuracy: 0.84, sensitivity: 0.99, specificity: 0.05) [42], the WHOOP Strap 3.0 (accuracy: 0.89, sensitivity: 0.95, specificity: 0.51) [43], and the Samsung Galaxy Watch (accuracy: 0.80–0.97) [44] were among the devices that demonstrated relatively satisfactory accuracy. The convenience of consumer sleep devices also simplified the collection of data from large-scale sleep samples on a global level. SCOTT H et al. [16] used the Withings Sleep Analyzer, a device placed under the mattress, to examine the relationship between sleep duration, irregular sleep patterns, and hypertension in a substantial global sample encompassing over 2 million nights. The researchers gathered data on TST, sleep onset, sleep midpoint, and sleep offset from 15,526 participants aged 18 to 90. Research proved that the equipment overestimated the TST of subjects by 25.8 min [19]. In contrast, the iSleep S200G and Sleep Dot B501 underestimated TST by 19.6 and 7.4 min, respectively. In another global study, researchers also employed the Withings Sleep Analyzer’s consumer user database to evaluate the prevalence, variability, and diagnostic misclassification of obstructive sleep apnea across multiple nights. This database included nightly sleep data from 87,610 participants collected from 1 July 2020 to 30 March 2021 [45].

In conclusion, the iSleep S200G and Sleep Dot B501 exhibited superior performance in accuracy evaluations relative to other consumer sleep devices, while also being cost-effective and user-friendly. They could address sleep monitoring requirements in diverse experimental contexts, including long-term, large-scale, or global sample populations. However, due to technical limitations, the accuracy of these two devices in detecting sleep is relatively low. Simultaneously, the iSleep S200G could not offer data on SOL or WASO. As a result, the device had to be chosen based on the experimental requirements. CCSTDs were an optional and easy experimental instrument for small-scale and short-duration studies. The measurement bias of these devices required critical evaluation. However, when performing large-scale, long-term sleep research on people who are unable or unwilling to wear sleep monitoring devices, relying on expensive PSG equipment and activity monitors for continuous monitoring was both impractical and challenging. In this situation, CCSTDs provided an innovative solution.

Currently, the rapid development of sleep-tracking technology has resulted in new devices and algorithms entering the market quicker than researchers could assess their performance [46,47]. We noted that the validation of some consumer sleep devices utilized in experimental investigations did not meet the established standards for validation. This issue encompassed the lack of key analyses of the ICC and EBE agreements. Lack of validation studies necessitated relying on validation data from older generations when employing later-generation devices. Researchers asserted that the new generation outperformed the previous generation.

The experimental results might have been affected by unpredictable errors, as the measurement bias between previous and current generations of devices was unclear. Consequently, it was imperative to implement standardized validation experiments prior to the implementation of the new device.

### 4.3. Limitations

The study had limitations. First, the experiment’s participants were limited to healthy young people. A more in-depth verification study was needed to ensure the accuracy of sleep monitoring in youngsters, older people who were more active during sleep, and individuals with sleep disorders. Second, while the sample size for this investigation was small, we rigorously vetted the subjects and sleep data to verify that they were representative and correctly reflected actual sleep patterns. Third, all of the individuals in this study slept alone, with no verification research conducted in settings including numerous beds in the same room or multiple people sleeping in the same bed. Finally, iSleep S200G and Sleep Dot B501 exhibited relatively low accuracy in monitoring light and deep sleep stages, and lacked the detection capability for the rapid eye movement sleep stage. Therefore, these two devices were not suitable for research requiring reliable sleep staging.

## 5. Conclusions

As representatives of the first generation of CCSTDs, the iSleep S200G and Sleep Dot B501 demonstrated reliable performance in monitoring sleep–wake states. In terms of EBE agreement and ICCs for TST, SE, SOL, and WASO, their performance was comparable to or even superior to that of actigraphy. Compared to PSG, the iSleep S200G showed no significant differences in TST and SE, whereas the Sleep Dot B501 demonstrated no significant differences in TST, SE, SOL, or WASO. Both devices showed greater agreement than actigraphy across these metrics. However, like other validated consumer-grade sleep monitoring devices, the iSleep S200G and Sleep Dot B501 exhibited relatively poor accuracy in sleep stage detection. Based on the validation results of this study, even early-developed CCSTDs could still meet the needs of long-term, large-scale preliminary or exploratory sleep research. However, the choice of device should depend on the required sleep monitoring indicators. The Sleep Dot B501 could monitor more sleep metrics than the iSleep S200G. Following the pattern of technological iteration, subsequent products generally outperform older versions. Therefore, their accuracy should not be worse than that of the two devices evaluated in this study. In addition to performance, cost is an important consideration. Long-term preliminary sleep monitoring does not rely on data from a single day. Higher precision is not always better, as it usually involves higher equipment costs. Therefore, a balance between performance and cost is necessary. Appropriate equipment should be selected based on the application’s requirements to ensure the study progresses smoothly.

## Figures and Tables

**Table 1:** A comparative analysis of the outcomes between PSG and each of the selected devices.

Outcomes	n	PSG Mean ± SD/ Med (IQR)	Device Mean ± SD/ Med (IQR)	Bias ^a^	LOA ^b^	t/w (*p*) ^c^	Effect Size	ICC ^e^ [95%CI] *p*
TST (min)								
iSleep ^f^	16	438.66 ± 65.90	420.94 ± 60.93	−17.72	−96.20, 60.76	1.77 (0.10)	0.28	0.78 [0.48–0.92] < 0.001
Sleep Dot ^g^	19	400.42 ± 98.20	391.45 ± 98.16	−8.97	−98.93, 80.99	0.85 (0.41)	0.09	0.89 [0.75–0.96] < 0.001
Actiwatch ^h^	16	399.25 ± 101.30	401.47 ± 70.60	2.22	−115.54, 119.98	−0.15 (0.88)	−0.03	0.77 [0.46–0.92] < 0.001
SE (%)								
iSleep	16	87.37% (75.65%, 92.78%)	85.14% ± 4.26%	1.36%	−15.36%, 17.96%	−0.16 (0.88) ^d^	−0.17	0.48 [−0.02–0.78] 0.03
Sleep Dot	19	84.40% (62.88%, 89.90%)	82.48% (71.15%, 89.23%)	1.92%	−18.80%, 22.64%	−0.44 (0.66) ^d^	−0.14	0.71 [0.40–0.88] < 0.001
Actiwatch	16	76.31% ± 15.24%	82.11% ± 8.66%	5.80%	−20.58%, 32.18%	−1.72 (0.11)	−0.47	0.38 [−0.07–0.72] 0.051
SOL (min)								
Sleep Dot	19	30.20 (20.10, 66.70)	49.00 (25.00, 66.00)	5.46	−57.91, 68.83	−0.78 (0.44) ^d^	−0.11	0.81 [0.57–0.92] < 0.001
Actiwatch	16	37.75 (19.80, 109.65)	60.66 ± 41.65	−4.71	−83.07, 73.65	−0.36 (0.72) ^d^	0.09	0.72 [0.36–0.89] 0.001
WASO (min)								
Sleep Dot	19	32.00 (18.10, 76.90)	28.52 (16.00, 53.64)	−3.96	−74.15, 66.23	0 (*p* > 0.99) ^d^	0.10	0.64 [0.27–0.84] 0.002
Actiwatch	16	33.70 (24.54, 70.13)	25.00 (14.25, 28.50)	−18.75	−78.92, 41.42	−2.07 (0.04) ^d^	0.53	0.56 [0.11–0.82] 0.004
Light Sleep (min)								
iSleep	16	255.44 ± 59.94	236.00 ± 25.57	−19.44	−129.57, 90.69	1.38 (0.19)	0.42	0.25 [−0.23–0.64] 0.16
Sleep Dot	19	224.18 ± 66.69	277.96 ± 75.38	53.78	−71.35, 178.91	−3.67 (0.002)	−0.76	0.47 [−0.01–0.77] 0.003
Deep Sleep (min)								
iSleep	16	41.09 ± 29.28	187.50 ± 50.75	146.41	44.65, 248.17	−11.28 (<0.001)	−3.53	0.03 [−0.04–0.18] 0.20
Sleep Dot	19	39.32 ± 27.27	112.49 ± 44.31	73.17	−15.83, 162.17	−7.02 (<0.001)	−1.99	0.08 [−0.09–0.35] 0.156

^a^ Bias: the mean differences between the test device and PSG. ^b^ LOA: limits of agreement (MD ± 1.96 SD). ^c^ t/w (*p*): paired *t*-tests or Wilcoxon signed-rank test. ^d^ Wilcoxon signed-rank test. ^e^ ICC: intraclass correlation coefficient. ^f^ iSleep: iSleep S200G. ^g^ Sleep Dot: Sleep Dot B501. ^h^ Actiwatch: Actiwatch Spectrum.

**Table 2:** EBE agreement: sleep versus wake.

Device	Accuracy	Sensitivity	Specificity	PPV	NPV
Actiwatch Spectrum	0.83	0.92	0.58	0.88	0.57
iSleep S200G	0.85	0.91	0.65	0.92	0.49
Sleep Dot B501	0.88	0.94	0.68	0.91	0.74

## Data Availability

The data that support the findings of this study are available from the corresponding author upon reasonable request.

## Abbreviations

The following abbreviations are used in this manuscript:

PSG	Polysomnography
CCSTDs	Contactless consumer sleep-tracking devices
TIB	Total time in bed
TST	Total sleep time
TWT	Total wake time
SE	Sleep efficiency
SOL	Sleep onset latency
WASO	Wake after sleep onset
N1	Light sleep
N2	Moderate sleep
N3	Deep sleep
REM	Rapid eye movement
EBE	Epoch-by-epoch
ICC	Intraclass correlation coefficient
LOA	Lower and upper limits of agreement
PPV	Positive predictive value
NPV	Negative predictive value
